# The leachate from the Urban Solid Waste Transfer Station produces neurotoxicity in Wistar rats

**DOI:** 10.1016/j.toxrep.2024.03.002

**Published:** 2024-03-08

**Authors:** Torres-González Omar Ricardo, Flores-Soto Mario Eduardo, Tejeda-Martínez Aldo Rafael, Sánchez-Hernández Iván Moisés, Chaparro-Huerta Verónica, Soria-Fregozo Cesar, González-Garibay Angélica Sofía, Padilla-Camberos Eduardo

**Affiliations:** a Unidad de Biotecnología Médica y Farmacéutica, Centro de Investigación y Asistencia en Tecnología y Diseño del Estado de Jalisco (CIATEJ), Mexico; b Division de Neurociencias, Centro de Investigación Biomédica de Occidente (CIBO), Mexico; c Departamento de Ciencias de la Tierra y de la Vida, Centro Universitario de los Lagos, Universidad de Guadalajara, Mexico

**Keywords:** Leachate, Landfill, In vivo, Neurotoxicity, Immunochemistry

## Abstract

Leachate from municipal solid waste is a mixture of xenobiotics capable of contaminating bodies of water and causing damage to the health of living beings that inhabit or consume contaminated water. A previous study revealed the presence of heavy metals in Urban Solid Waste Transfer Station (USWTS) leachate above the permissible national and international limits. In the present study, we demonstrate that subchronic oral administration (5 and 25 % v/v) of leachate to male Wistar rats caused changes in the immunoreactivity of the glial markers: GFAP and Iba-1, accompanied by an increase in the expression of caspase-3, and a decrease in the expression of the NeuN protein. Results indicate that the heavy metals present in the leachate induced neuronal loss in the prefrontal cortex, suggesting that these contaminants can cause neurological problems in mammals that consume surface water with xenobiotics, since the leachate could contaminate water bodies and underground water.

## Highlights

• Subchronic oral administration of leachate to male Wistar rats caused neurotoxicity.
• Oral administration of leachates changes the immunoreactivity of GFAP and Iba-1.
• Oral leachates administration increases the expression of caspase-3 and decreases the expression of the NeuN protein.
• Heavy metals of a leachate cause neurological problems in mammals that consume contaminated water.

## 1 Introduction

Waste is a direct result of anthropogenic activity, and its confinement represents a public health problem, the degradation of liquid, solid and gaseous waste produces substances that pollute the environment and directly affect human health [42], [50]. Composition of the landfill leachate can vary depending on the chemical compound of the waste, the type of storage system, and the environmental conditions of the final disposal site [44]. Generally, landfill leachate is a brown, liquid, heterogeneous mixture of organic compounds, salts, and heavy metals [4], [18]. A few reports have shown that xenobiotics present in landfill leachate induce alterations in the structure and function of organs such as the liver and kidney, and genetic alterations in blood cells and bone marrow of rodents experimentally exposed by oral route [3], [27], [45].

The mechanisms whereby environmental xenobiotics such as heavy metals produce negative effects on the central nervous system are widely known; however, few studies have demonstrated the synergistic effects of such pollutants contained in landfill leachate in the environment on mammalian nervous systems. For example, Li, et al. [27] showed that 7 days of oral exposure to drinking water from leachate obtained from a landfill in Xingou (Taiyuan, China) caused neuronal damage due to oxidative stress through lipid peroxidation in the liver and brain of Kunming mice of both sexes. The authors measured and verified the synergy of environmental pollutants causing alterations in the antioxidant action system, such as the enzymatic activity of copper- and zinc-superoxide dismutase (Cu/Zn SOD), glutathione peroxidase (GPx), and catalase (CAT). Alimba, et al. [4] demonstrated that in male Wistar rats, oral exposure to different concentrations of leachate obtained from the Olusosun and Aba-Eku landfills in Nigeria induced brain damage through oxidative stress, suggesting that environmental contaminant conditions were present, and the leachate was a risk to wildlife and humans.

For their part, Adeniyi, et al. [1] measured neurodegeneration in the prefrontal cortex of adult male Wistar rats induced by heavy metals present in fluvial electronic waste (e-waste) from Nigeria. The e-waste with higher concentrations of heavy metals was administered ad libitum for 65 days. They demonstrated that oral exposure to e-waste produced oxidative damage in the prefrontal cortex of treated animals compared to the control group. In addition, a previous work demonstrated that exposure to 10 % leachate from the Gwagwalada, Nigeria, landfill for 21 days (oral route), altered both body and brain weight, accompanied by an increase in the loss of Purkinje cells and an increase in glial reactivation (microglia cells and astrocytes) [26]. However, the mechanism of action by which these alterations occur is unknown to date.

Previously, it was found that the leachates from the Urban Solid Waste Transfer Station (USWTS) of the city of Guadalajara, Jalisco, Mexico, contained heavy metals such as mercury (Hg) above the permissible limits established by the official Mexican standard NOM-002-SEMARNAT-1996 (NOM-002-ECOL-1996) [38], which establishes the maximum permissible limits of contaminants in wastewater discharges to urban or municipal sewage. The same study found that the leachate also surpassed the U.S. Environmental Protection Agency (US-EPA) standards for arsenic (As), cadmium (Cd), chromium (Cr), mercury (Hg), and lead (Pb). Oral exposure to the leachate induced genotoxicity and cytotoxicity in peripheral blood erythrocytes of pre-adolescent and young adult male Wistar rats after a 30-day subchronic toxicity test [45].

It is known that in mammals, brain areas such as the prefrontal cortex are target regions for environmental pollutants such as heavy metals [22], [49]. Therefore, in the present work, we exposed male Wistar rats to USWTS leachate subchronically and evaluated the effect on neurons of the prefrontal cortex based on reactivity to NeuN (exclusive nuclear marker of neurons), GFAP (acid gliofibrillary protein, essential component of the cytoskeleton of astrocytes), Iba-1 (a microglia/macrophage-specific calcium-binding protein), and caspase-3 (key effector enzyme in the induction of cell apoptosis). Understanding the effect of leachate on this brain area is important, since it plays a critical role in the mood sensory-motor and cognitive processes of mammals, and when affected by environmental pollutants could be the cause of neurological diseases in humans.

## 2 Materials and methods

The study site, the method of obtaining leachate, and the physicochemical characterization were previously described [45]. The leachate was filtered with 0.2 µM Whatman® filter paper (MAIDSTONE, UK) to remove the supernatant. The leachate was centrifuged (Centrifuge 5804, EPPENDORF, GERMANY) at 3000×*g* for 10 min; the supernatant was considered the 100 % stock sample and stored at 4 °C until use. For the biological test, two concentrations of leachate LCH5 % v/v and LCH25 % v/v in distilled water were prepared. Concentrations were selected based on the results of the neurotoxic effect of different leachates found by [4]

### 2.1 Physicochemical analysis of the leachate

The physicochemical characterization of the leachate was carried out at the Unidad de Servicios Analíticos y Metrológicos of the Centro de Investigación y Asistencia en Tecnología y Diseño del Estado de Jalisco (CIATEJ). The following physicochemical parameters were determined in accordance with the maximum permissible limits of contaminants specified in the official Mexican standard NOM-002-SEMARNAT-1996 (NOM-002-ECOL-1996) [38], which establishes the maximum permissible limits of pollutants in wastewater discharges to urban or municipal sewage systems, and the international US-EPA standard: pH, temperature, biochemical oxygen demand (BOD) and total nitrogen (NT).

### 2.2 Biological analysis

#### 2.2.1 Animals

For this experiment, fifteen male Wistar rats of 90-days-old were provided by the Centro de Investigación Biomédica de Occidente (CIBO), Instituto Mexicano Del Seguro Social (IMSS). The animals were housed in translucent acrylic boxes (44 × 33 × 25 cm) in a room at room temperature, with a light/dark cycle of 12/12 h (the light was turned on at 7:00 am) and *ad libitum* access to water and rodent breeding diet pellets A-30 (SAFE, FRANCE). Animals were handled following the animal care guidelines of the official Mexican standard (NOM-062-ZOO-1999) [39], which establishes the technical specifications for the production, care and use of laboratory animals, and the study was approved by the CIBO-IMSS Ethics and Health Research Committee with registration number R-2019–1305–6.2.5.

### 2.3 Experimental design

The animals were randomly distributed into 3 groups, n=5 for each group. A concentration of 5 % landfill leachate (LCH5 %) was administered to the first group, and 25 % landfill leachate (LCH25 %) was administered to the second group. The third group was administered with distilled water (negative control). In total, 500 µL of each treatment was administered to each animal orally with a stainless steel cannula for 30 consecutive days according to Alimba et al. [4]. The volume dose was adequate for the administration of substances to laboratory animals [47]

### 2.4 Immunohistochemistry

The procedure was performed under the same conditions reported by Halene et al. [19]. At the end experimental assay, animals from all groups were euthanized by decapitation and the brain was removed, and fixed in 4 % formaldehyde (SIGMA, USA) in 0.1 M phosphate buffer PBS (SIGMA, USA). Sections of the prefrontal cortex were made with a vibratome (LEICA VT1000S, USA) and stored in a solution of 1X PBS and 0.1 % sodium azide (AFFYMETRIX, USA) for later use. To perform immunohistochemistry, the tissue obtained was washed three times with 1X PBS, then incubated in a borohydrate solution for 10 minutes. After that, several washes were performed with 1X PBS until the borohydrate was completely eliminated. Subsequently, the tissue was incubated with agitation in a solution of 1X PBS solution with 0.5 % Triton (SIGMA, USA) and sodium azide for 1 hour. To remove the previous solution, three washes were carried out with 1X PBS. After this, the tissue was incubated in a solution of 1X PBS and 0.3 % H_2_O_2_ (SIGMA, USA) for 10 minutes. After that, several washes were performed to remove the solution with 1X PBS, 0.1 % Triton, and 0.1 % sodium azide. The tissue was incubated in 1:100 equine serum (MERCK, GERMANY) for 30 minutes prior to incubating for 24 h at 4 °C with anti-Caspase 3, (1:500; ABCAM) anti-NeuN (1:1000; ABCAM, UK), anti-GFAP (1:1000; ABCAM, UK), anti-Iba-1 (1:1000; ABCAM, UK) the antibodies were diluted in 1X PBS. After washing in 0.1 M PBS, antibody binding was detected by incubating for 2 h at room temperature in the dark with a biotinylated anti-mouse IgG (BA1000, VECTOR LABS, UK) or anti-rabbit IgG (BA5000, VECTOR LAB, UK) antibody, both diluted 1:500. After washing, the Elite ABC kit (PK6000, VECTOR LAB, UK,) was used with 3,3’-diaminobenzidine (VECTOR LAB, UK) to visualize the antibodies. The average number of labelled cells per field was evaluated at a 40x magnification of the tissue.

### 2.5 Immunostaining analysis

The analysis was carried out in the prefrontal cortex. A total of two fields per tissue were analyzed with a distance of 40 μm between them with a total increase of 40x. Photograph of each of the previously described fields was taken using the 5.0-megapixel Moticam camera and the Motic images plus 2.0 software. Using the free-use software ImageJ 1.5e (WAYNE RASBAND, USA) the positive area for NeuN staining of each photograph was quantified. Finally, the “Measure” function was used to quantify staining intensity in each image, expressed as arbitrary units of intensity. For the analysis of GFAP, caspase-3, and Iba-1, each of the previously described fields was quantified and the total number of positive cells per field was quantified.

### 2.6 Statistical analysis

Data are presented as the mean ± SEM. All data was analyzed using GraphPad Prism 8.0.1 software (CALIFORNIA, USA). All data obtained were analyzed using a 1-way ANOVA. It was performed as Dunnett's *post-hoc* when the value of p≤0.05.

## 3 Results

### 3.1 Physicochemical analysis of the leachate

Table 1 shows the physicochemical parameters analyzed of the USWTS of Guadalajara Jalisco, Mexico leachates. Leachate landfill was brown color, with acid pH, temperature, BOD, and total nitrogen values within specifications of NOM-002-SEMARNAT-1996 (NOM-002-ECOL-1996) [38], which establishes the maximum permissible limits of pollutants in wastewater discharges to urban or municipal sewage systems, and US-EPA.

**Table 1:** Physicochemical analysis of the leachate of USWTS.

**PARAMETER**		**NOM 002-ECOL-1996**	**US-EPA**
**pH**	3–6	5.5–10	6.5–8.5
**TEMPERATURE**	42	40	-
**BOD**	26,746.72	200	-
**TOTAL NITROGEN**	1606.94	200	-

The pH values are represented as a logarithmic scale of hydrogen potential. Temperature is expressed as Celsius degrees. BOD and total nitrogen are presented as mg/L.

#### 3.1.1 Immunohistochemistry of NeuN, Caspase-3, GFAP and Iba-1

##### 3.1.1.1 Expression of NeuN

**Fig. 1:**
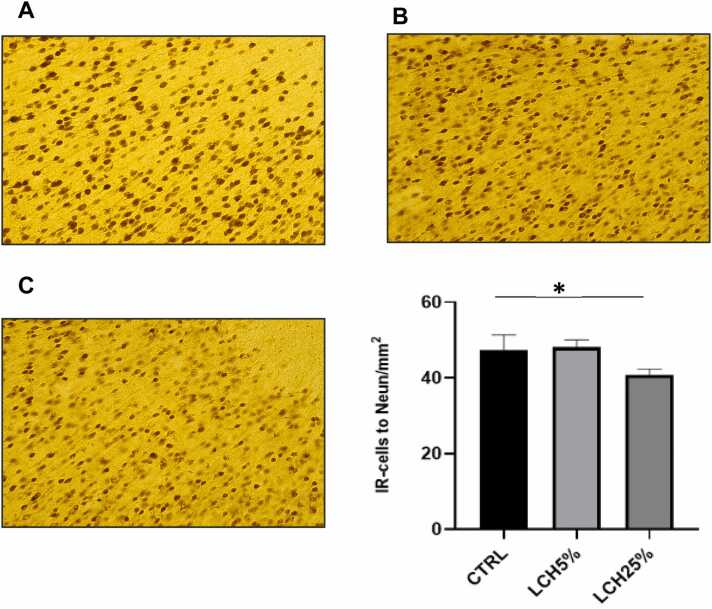
NeuN expression in prefrontal cortex of control and of animals administered orally with LCH5 % and LCH25 %. (A) Control, (B) LCH5 % and (C) LCH25 %. The cells are shown in higher magnification. (D) Quantification of NeuN immunoreactivity was expressed as the total number of positive cells per field in the prefrontal cortex. The number of animals was 5 for each group. Data are means ± SEM. * p < 0.05, control *vs* LCH25 %.

The results of the present work showed that the oral administration of the leachate at a concentration of 25 % (p<0.01) caused a decrease in the number of cells positive for NeuN when compared with the control group. It is worth mentioning that the dose of 5 % of the leachate did not cause a decrease in the immunoreactivity of NeuN compared with the control group (Fig. 1).

### 3.2 Expression of Iba-1, GFAP and Caspase-3

**Fig. 2:**
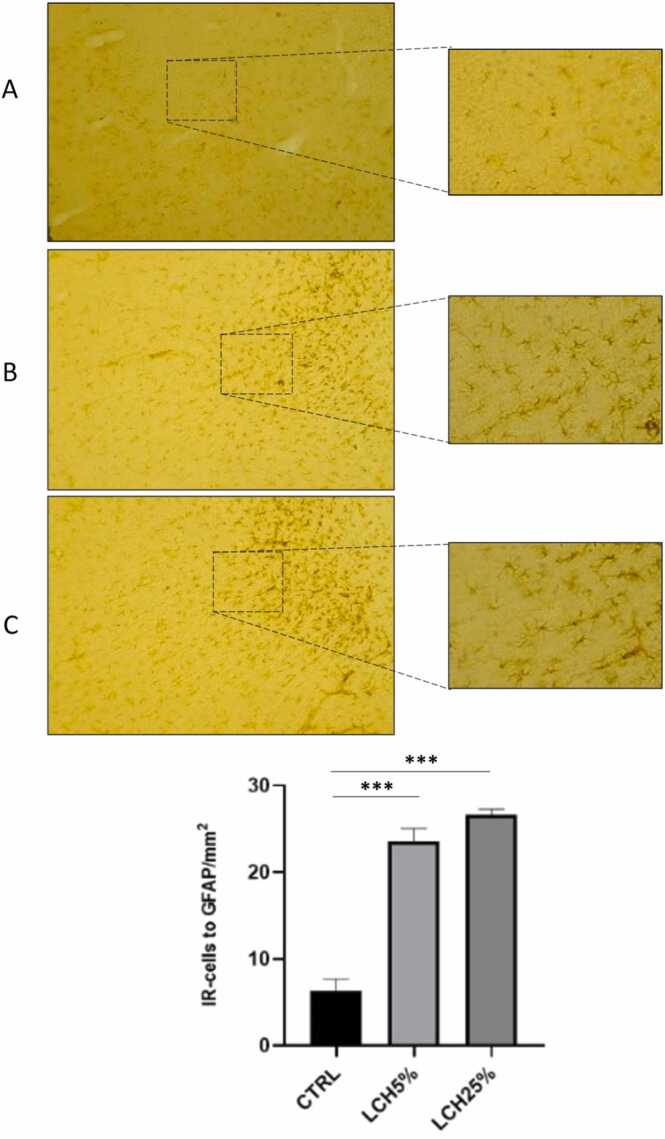
Astrocyte (GFAP) expression in prefrontal cortex of control and of animals administered orally with LCH5 % and LCH25 %. (A) Control, (B) LCH5 % and (C) LCH25 %. The cells pointed to by arrows are shown in higher magnification. (D) Quantification of GFAP immunoreactivity was expressed as the total number of positive cells per field in the prefrontal cortex. The number of animals was 5 for each group. Data are means ± SEM. *** p < 0.001, control *vs* LCH5 %. *** p < 0.001, control *vs* LCH25 %.

**Fig. 3:**
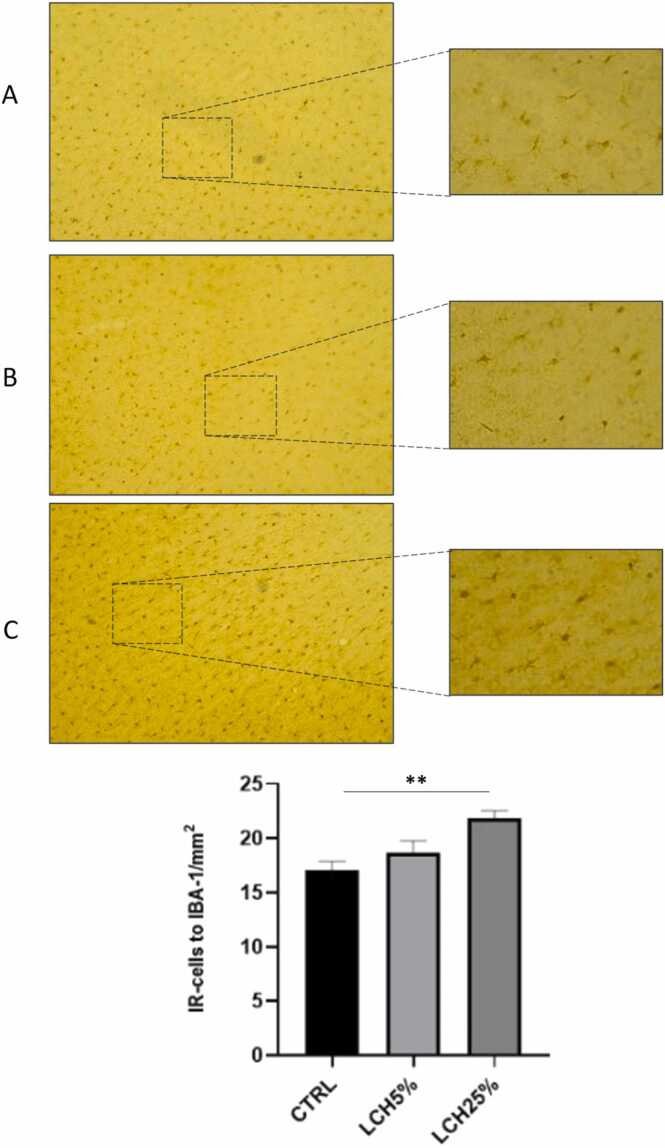
Microglia (Iba-1) expression in prefrontal cortex of control and of animals administered orally with 5 and 25 % leachate. (A) Control, (B) LCH5 % leachate and (C) LCH25 % leachate. The cells pointed to by arrows are shown in higher magnification. (D) Quantification of iba-1 immunoreactivity was expressed as the total number of positive cells per field in the prefrontal cortex. The number of animals was 5 for each group. Data are means ± SEM. ** p < 0.01, control vs. 25 % leachate.

Regarding the immunoreactivity of the glial cells, the oral administration of the leachate at a concentration of 25 % increased the immunoreactivity of the cells positive for GFAP and Iba-1 with respect to the control group (Fig. 2, Fig. 3). However, the 5 % concentration of the leachate caused only an increase in the number of GFAP-positive cells (Fig. 2).

**Fig. 4:**
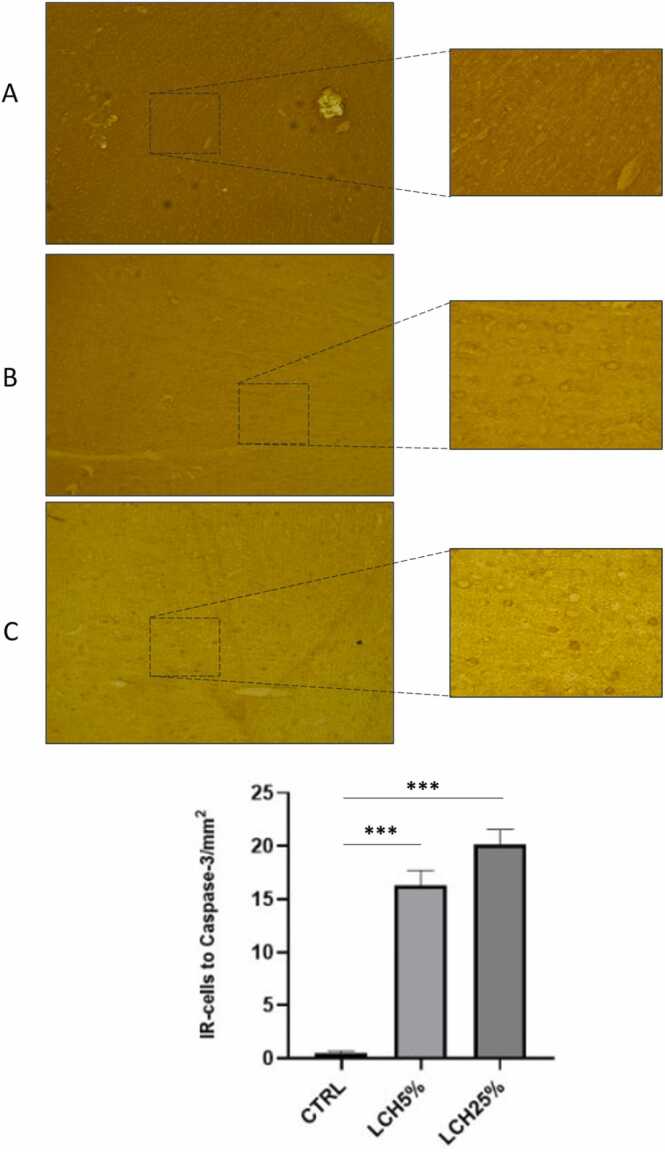
Caspase-3 expression in prefrontal cortex of control and of animals administered orally with LCH5 % and LCH25 %. (A) Control, (B) LCH5 % and (C) LCH25 %. The cells pointed to by arrows are shown in higher magnification. (D) Quantification of caspase-3 immunoreactivity was expressed as the total number of positive cells per field in the prefrontal cortex. The number of animals was 5 for each group. Data are means ± SEM. *** p < 0.001, control *vs* LCH5 %. *** p < 0.001, control *vs* LCH25 %.

On the other hand, the oral administration of the leachate at a concentration of 5 and 25 % caused an increase in the number of caspase-3 positive cells with respect to the control group (Fig. 4).

## 4 Discussion

In the work that preceded this study, it was determined that leachate obtained from USWTS contained Hg (0.10 mg/L) above the permissible limits of the official Mexican standard (NOM-002-ECOL-1996) [38] and concentration of As (0.010 mg/L) Cd (0.025 mg/L), Cr (1.00 mg/L), Hg (0.10 mg/L), and Pb (1.00 mg/L) above the permissible limits of USEPA [45]. In the present study, we administered USWTS leachate orally to male Wistar rats and evaluated the neurotoxic effect of the leachate on the prefrontal cortex through the evaluation of the expression of glial immunoreactivity, NeuN and a marker of apoptotic cell death caspase-3. We observed that the doses used (25 % v/v) decreased the reactivity of the NeuN protein and caused an increase in glial cell immunoreactivity and caspase-3.

The mechanism of action by which the leachate causes these neurotoxic effects is currently unknown, however, it has been proposed that this effect is caused by the high content of metals present in the leachate. The studies carried out by Niño, et al. [37] evaluated chronic As exposure in male and female adolescent and adult Wistar. The study indicated that As exposure was associated with a negative and persistent impact on the microstructural characteristics of white matter tracts after 12 months. Other study has shown that exposure to Cd significantly affects peripheral nervous system (PNS) and central nervous system (CNS) function, with a wide spectrum of clinical symptoms including olfactory dysfunction, peripheral neuropathy, neurological abnormalities, mental retardation and learning disabilities, impaired motor activity, and behavioral disturbances in both adults and children [33]. These effects are related to structures such as the prefrontal cortex [9]. Cd can be inhaled and easily distributed within the CNS in young individuals [34]. Regarding the Cd exposure, López, et al. [29] demonstrated apoptosis induction in neurons by generating reactive oxygen species (ROS), inducing mitochondrial membrane dysfunction, and reducing adenosine triphosphate (ATP) levels [48]. In addition, Cd exposure triggered a necrotic process in neurons due to lipid peroxidation and changes in the activation of antioxidant enzymes. Similarly, Hg alters the mitochondrial function and activity of the enzyme ATP-ase Na^+^/K^+^ by generating ROS and affecting the CNS [32]. On the other hand, both acute and chronic exposure to Pb induce cell death in the prefrontal cortex of rats, altering brain function through oxidative stress and enzyme inhibition, which promotes decreased reactivity of NeuN protein [9], [10], [31], [32], [36]. Pb alters the development of the nervous system in mammals and damages other organs [14]. Regarding Cr, it is known that it is required in trace amounts for lipid and protein metabolism, as well as for insulin action. Nevertheless, Cr has multiple oxidation states, which modify the risk of its bioaccumulation in the human body and can cause neurological diseases, among other health issues [7]. The higher concentration of the above-mentioned elements present in the leachate evaluated in this study might explain the effects in the prefrontal cortex of exposed rats. In this work, a significant increase in the levels of BOD demand was found. Generally, the leachate from a landfill contains a large number of organic products, heavy metals, high ammonia content and, as it matures, there is a transforming effect on the organic substances present [41], [43] limiting free oxygen necessary to transform organic matter through aerobic microbial decomposition [28], [8]. If leachate high in BOD is released into a body of water, it can affect aquatic life and subsequently affect water for human consumption [12].

In addition, it has been established that an increase in BOD is related to the presence of bacteria with the capacity to bio-transform environmental pollutants such as Hg [15]. Feng et al. demonstrated that the mercury methylation process occurs under aerobic conditions and is dependent on the concentration of dissolved oxygen in artificial reservoirs, since their physicochemical conditions are conducive to easy contamination, increasing the bioavailability, methylation, and bioaccumulation of Hg [11]. In our findings, BOD is elevated, which would maintain the environment conducive to the biotransformation and bioamplification of heavy metals due to microbial activity.

Of all the chemical forms of Hg, methylmercury (MeHg) is the most toxic, being highly lipid soluble, which allows it to cross plasma membranes and affect the permeability of the placental and blood-brain barriers. Among the effects that have been reported are loss of speech, hearing, visual, dementia, and dysarthria. In addition, MeHg has been shown to cause an increase in pro-inflammatory processes in the brain, through the activation of microglia cells [40]. In this sense, [46], demonstrated that MeHg induced an increase in the expression of TNF-ɑ mediated by microglia cells, through the mtROS/ASK1/p38 pathway, causing an increase in the levels of ROS, therefore, it is possible that one of the mechanisms by which MeHg induces neuronal damage and death is through an increase in the oxidizing environment accompanied by a decrease in antioxidant capacity at the brain level, which leads to neuronal damage and death by apoptosis (activation of caspases). Although MeHg was not measured, the conditions for its production seem favorable, so the presence of Hg in the USWTS leachate could be related to the increased expression of caspase-3, as low (5 %) concentrations administrated and higher dose (25 %) increased the expression of caspase-3.

Another interesting result found in this study was the high level of nitrogen levels and a decrease in pH. It is important to mention that nitrogen in leachates is found as ammonia (NH_3_) and as ammonium (NH_4_). When these are dumped into bodies of water, as the pH and temperature increase, there will be a greater presence of ammonia, however, if the pH drops, there will be a greater presence of ammonium ion. It is important to mention that both elements are toxic, both to humans and to some species of animals due to their ability to cross cell membranes, in addition, nitrites and nitrates can be formed from ammonium, and it is well known that these compounds are highly toxic, an example of this is that a high concentration of nitrates in drinking water or its consumption causes methemoglobinemia in infants and stomach cancer in adults this results in the development of cyanosis, stupor, and cerebral anoxia [5].

Regarding the results obtained in the evaluation of NeuN protein reactivity in neurons of the prefrontal cortex of rats exposed to leachate for 30 days, a statistically significant decrease in NeuN protein expression was observed with a leachate concentration of 25 % v/v. It should be considered that while healthy neurons show intense NeuN expression, decreased NeuN positivity in post-embryonic life may be indicative of the degeneration of differentiated neurons [35]. Immunoreactivity is significantly weakened after severe injury, such as hypoxia or cerebral ischemia [25], an effect presumably caused by the combined environmental contaminants present in the leachate. Studies have shown that damaged neurons can reduce NeuN protein expression by reverting to a less mature state that allows the nervous system development [20].

There are reports supporting the effects of environmental pollutants such as heavy metals on terrestrial mammalian animals such as rats, mice, and even humans at different ages, with direct effects on the function of organs involved. For decades, it has been debated whether environmental pollutants have a greater effect on the first stages of the mammal life cycle. It is known that certain stages of development are critical, so evaluating the possible effects of exposure in terms of the chemical form, dose, or route of administration of a metal is extremely difficult [2], [23]. Animals in advanced stages or senescence also often show increased susceptibility to the effects of these contaminants on organs [16].

There is little information on the synergistic effects of environmental pollutants present in mixtures produced by the degradation of garbage; the nature of leachates relies on the characteristics of urban solid waste, and phenomena such as technological blackouts and an insufficient culture of recycling increase the presence of emerging and re-emerging toxins that will presumably pose a very high risk to human and environmental health [13], [17]. Synergy occurs when the interaction between two or more pollutants makes the total effect greater than the individual effect. These mechanisms are often underestimated and can lead to both a decrease and an increase in the summary toxicity of xenobiotic cocktails in exposed organisms [24].

We demonstrate that the USWTS leachate decreased cell population in the prefrontal cortex of pre-adolescent and young adult rats after oral exposure to concentrations of 5 and 25 % v/v, as reported by [4] where they found brain damage using those concentrations of two different leachates. Although, the real human risk assessment of the USWTS leachate evaluated in the present study remains to be determined, it is known that the composition of leachate is heterogeneous, and heavy metals, as well as many other compounds, might be present [4], [6]. Some authors have utilized formulas acknowledging heavy metals for hazard quotient [21], but many other characteristics of leachate -such as dissolved organic matter, microplastics, and macro inorganic materials- might change the risk. Additionally, the risk to human health is related to those compounds found as pollutants in drinking water or in the food chain -livestock and agriculture-, air and soil, depending on the proximity of human settlements to the landfill [18]. The leachate may leak into groundwater aquifers due to rainfalls, spread into the adjacent river system by groundwater flow, and pollute the surrounding environment [30].

We found higher immunoreactivity of the cells positive for GFAP and Iba-1 when administering 25 % concentrate of leachate, this coincides with a recent study showing an increase in immunoreactivity for GFAP (positive astrocytes) and Iba-1 accompanied by hypertrophy of microglial cell body when Wistar rats received 10 % of leachate [26].

## 5 Conclusions

Leachate obtained from the USWTS at doses of 5 and 25 % induced changes in the immunoreactivity of the glial markers: GFAP and Iba-1, accompanied by an increase in the expression of caspase-3 and a decrease in the expression of the NeuN protein of the prefrontal cortex of male Wistar rats during subchronic exposure of 30 days, associated with the heavy metal content of the leachate. Heavy metals, even at low concentrations, induce the development of pathological processes in chronically exposed organisms. The synergistic action of heavy metals causes negative neurological effects in mammals that consume water contaminated with xenobiotics in leachate (deliberately or accidentally) released into surface and groundwater bodies.

## CRediT authorship contribution statement

**Eduardo Padilla:** Supervision, Project administration, Funding acquisition, Conceptualization. **González-Garibay Angélica Sofía:** Writing – review & editing, Visualization. **Soria-Fregozo Cesar:** Supervision, Formal analysis, Data curation, Conceptualization. **Chaparro-Huerta Verónica:** Supervision, Conceptualization. **Sánchez-Hernández Iván Moisés:** Writing – review & editing, Data curation. **Tejeda-Martínez Aldo Rafael:** Writing – original draft, Formal analysis. **Flores-Soto Mario Eduardo:** Supervision, Conceptualization. **Torres-González Omar Ricardo:** Methodology, Investigation.

## Declaration of Competing Interest

The authors declare that they have no known competing financial interests or personal relationships that could have appeared to influence the work reported in this paper.

## Data Availability

Data will be made available on request.
